# The expression of S100A8/S100A9 is inducible and regulated by the Hippo/YAP pathway in squamous cell carcinomas

**DOI:** 10.1186/s12885-019-5784-0

**Published:** 2019-06-17

**Authors:** Yunguang Li, Fei Kong, Chang Jin, Enze Hu, Qirui Shao, Jin Liu, Dacheng He, Xueyuan Xiao

**Affiliations:** Key Laboratory of Cell Proliferation and Regulation Biology, Ministry of Education, Beijing Normal University, 19th, Beijing, 100875 China

**Keywords:** S100A8/S100A9, Co-expression and co-localization, Hippo pathway, YAP, F-actin, Proliferation and differentiation, Cell apoptosis

## Abstract

**Background:**

S100A8 and S100A9, two heterodimer-forming members of the S100 family, aberrantly express in a variety of cancer types. However, little is known about the mechanism that regulates S100A8/S100A9 co-expression in cancer cells.

**Methods:**

The expression level of S100A8/S100A9 measured in three squamous cell carcinomas (SCC) cell lines and their corresponding xenografts, as well as in 257 SCC tissues. The correlation between S100A8/S100A9, Hippo pathway and F-actin cytoskeleton were evaluated using western blot, qPCR, ChIP and Immunofluorescence staining tests. IncuCyte ZOOM long time live cell image monitoring system, qPCR and Flow Cytometry measured the effects of S100A8/S100A9 and YAP on cell proliferation, cell differentiation and apoptosis.

**Results:**

Here, we report that through activation of the Hippo pathway, suspension and dense culture significantly induce S100A8/S100A9 co-expression and co-localization in SCC cells. Furthermore, these expressional characteristics of S100A8/S100A9 also observed in the xenografts derived from the corresponding SCC cells. Importantly, Co-expression of S100A8/S100A9 detected in 257 SCC specimens derived from five types of SCC tissues. Activation of the Hippo pathway by overexpression of Lats1, knockdown of YAP, as well as disruption of F-actin indeed obviously results in S100A8/S100A9 co-expression in attached SCC cells. Conversely, inhibition of the Hippo pathway leads to S100A8/S100A9 co-expression in a manner opposite of cell suspension and dense. In addition, we found that TEAD1 is required for YAP-induced S100A8/S100A9-expressions. The functional studies provide evidence that knockdown of S100A8/S100A9 together significantly inhibit cell proliferation but promote squamous differentiation and apoptosis.

**Conclusions:**

Our findings demonstrate for the first time that the expression of S100A8/S100A9 is inducible by changes of cell shape and density through activation of the Hippo pathway in SCC cells. Induced S100A8/S100A9 promoted cell proliferation, inhibit cell differentiation and apoptosis.

**Electronic supplementary material:**

The online version of this article (10.1186/s12885-019-5784-0) contains supplementary material, which is available to authorized users.

## Background

Approximately 80% of human cancers are epithelial in origin, with SCC being one of the most common tumor types. SCCs predominantly derived from the squamous epithelia of the skin, oral cavity, esophagus, and cervix and can be highly aggressive and metastatic [1]. Despite surgery, radiotherapy, and chemotherapy, SCC lesions often recur and spread to other body sites, such as the lung. Therefore, it is important to identify the molecules that inhibit the aberrant proliferation of SCC, as it may help in improve the clinical treatments of SCCs.

S100A8 (calgranulin A) and S100A9 (calgranulin B), two heterodimer-forming members of the S100 family, were originally discovered as immunogenic proteins expressed and secreted by neutrophils [2]. Additionally, constitutive expression of S100A8/S100A9 is largely restricted to myeloid cells and tightly regulated during myeloid differentiation [3–5]. In phagocytic and nonphagocytic cells, S100A8 and S100A9 form heteromeric complexes that bind arachidonic acid (AA) and promote NADPH oxidase activation [6]. Therefore, S100A8/S100A9 have emerged as important pro-inflammatory mediators of acute and chronic inflammation [7]. Although researchers focused on the roles of S100A8/S100A9 in inflammatory cells currently, there is also growing evidence for important roles of both proteins in non-myeloid cells, such as skin cells and cancer cells [8, 9]. In normal skin, S100A8/S100A9 minimally express, but they are massively expressed in psoriasis keratinocytes, which demonstrate abnormal differentiation and hyperproliferation [10, 11]. Aberrant expression of S100A8/S100A9 complex was also detected in a variety of cancer tissues, including in squamous cell carcinomas of the esophagus, oral cavity, and cervix [2, 12–15]. It has reported that strong expression and secretion of S100A8/S100A9 may be associated with the loss of estrogen receptor in breast cancer, and may be involved in the poor prognosis of Her2+/basal-like subtypes of breast cancer [16]. S100A8/S100A9 expression in epithelial cancer cells causes enhanced infiltration of immune cells, especially neutrophils, and stimulates settlement of the cancer cells in the lung [17]. Some work demonstrated extracellular S100A8/S100A9 proteins contribute to colorectal carcinoma cell survival and migration via Wnt/β-catenin pathway [18]. However, few studies have investigated whether S100A8/S100A9 displayed co-expressions in SCC tissues and how these two proteins regulated in SCC cells.

The Hippo/YAP-signaling pathway is a critical regulator of tissue homoeostasis [19]. At the core of this pathway in mammals includes MST1/2, and their substrates, the kinases LATS1/2 [20]. LATS1/2 (LATS-HM) is phosphorylated by MST1/2, which in turn directly phosphorylates YAP (Yes-associated protein) at Serine 127 (YAP-S127). The phosphorylated YAP results in its cytoplasmic localization and no longer acts as a transcriptional coactivator [20–24]. However, dephosphorylated YAP exerts its transcriptional activity mostly by interacting with the TEAD in nucleus [24]. TEADs are the primary transcription factors and play an important role in development, tissue homeostasis, and tumorigenesis. Importantly, these functions of TEADs including cell proliferation, differentiation, and survival are largely thought to be regulated by binding of YAP/TAZ [25]. Interestingly, recent studies demonstrate that changes of cell shape and/or cell density also activate the Hippo-YAP pathway via actin cytoskeleton reorganization [26, 27].

In this study, we found that both S100A8 and S100A9 are inducible under cell suspension and dense culture through activation of the Hippo pathway in SCC cells. Consistent with these findings, S100A8/S100A9 co-localization and/or co-expression also found in SCC cells and their corresponding xenografts, as well as in the clinical SCC tissues. We also proved that S100A8/S100A9 functions as promoting cell proliferation, but inhibiting squamous differentiation and apoptosis induced by suspension and dense culture. Our findings provide new insights into the Hippo/YAP pathway in regulation of S100A8/S100A9 expression in SCCs.

## Methods

Detailed methods include Plasmids and Reagents, siRNA and transfection, RT-PCR, Immunofluorescence staining, Tumorigenicity in nude mice, Tissue specimens, ChIP, Flow Cytometry are in Additional file 1.

### Cell culture

The human carcinoma cell lines A431, HCC94, and FaDu were purchased from the Chinese Academy of Sciences Committee Type Culture Collection Cell Bank and the cell lines were authenticated by short tandem repeat analysis at HK Gene Science Technology Co (Beijing, China). All cells were cultured in 1640 medium with 10% fetal calf serum. Cells were maintained in a humidified incubator at 5% CO_2_ and 37 °C. Suspension culture was achieved by plating the cells in Poly-HEMA coated (12 mg/ml dissolved in 95% ethanol) 6-well plates in medium. Low cell density (here-after called ‘sparse’) were achieved by plating 10,000 cells/cm^2^ in plates, and high cell density (here-after called ‘dense’) were achieved by plating 100,000 cells/cm^2^ in plates.

### Western blot

Western blotting analysis performed as previously described [28, 29]. The following antibodies were used: S100A8 (1/1000; R&D, MAB4570); S100A9 (1/1000; Santa-Cruz,8114); MST1 (1/1000; Cell Signaling Technology, 3682, Boston, USA); LATS1 (1/1000; Cell Signaling Technology, 3477); pLATS1-T1079 (1/1000; Cell Signaling Technology, 8654); YAP (1/1000; Cell Signaling Technology, 4912); pYAP-S127 (1/1000; Cell Signaling Technology, 13008S); anti-Flag tag (CWBIO, CW0287A, Beijing, China); anti-His tag (MBL, D291–3, NAGOYA, JAPAN). GAPDH (ZSGB-BIO, TA-08, Beijing, China) was used as loading control.

### Immunohistochemistry

Immunohistochemistry performed as described in our previous study [30]. Anti-S100A8 (1:150) and anti-S100A9 (1:150) separately incubated with the specimens. The goat anti-Mouse (KIT-5010) and Rabbit-anti goat (KIT-5017) secondary antibody purchased from MAIXINBIO (Fujian, China). S100A8 and S100A9 expression detected using a light microscope (ZEISS ImagerA1).

### Statistical analysis

Statistical analysis performed using GraphPad Prism software. The statistical significance evaluated using Student’s t-test (2-tailed) to compare two groups of data. The asterisks indicate significant differences between the experimental groups and corresponding control condition. Differences considered statistically significant at a *P*-value of less than 0.05. *P-*values < 0.05, < 0.01 are indicated with one and two asterisks, respectively.

## Results

### S100A8/S100A9 is inducible and both proteins display co-localization in vitro

**Fig. 1:**
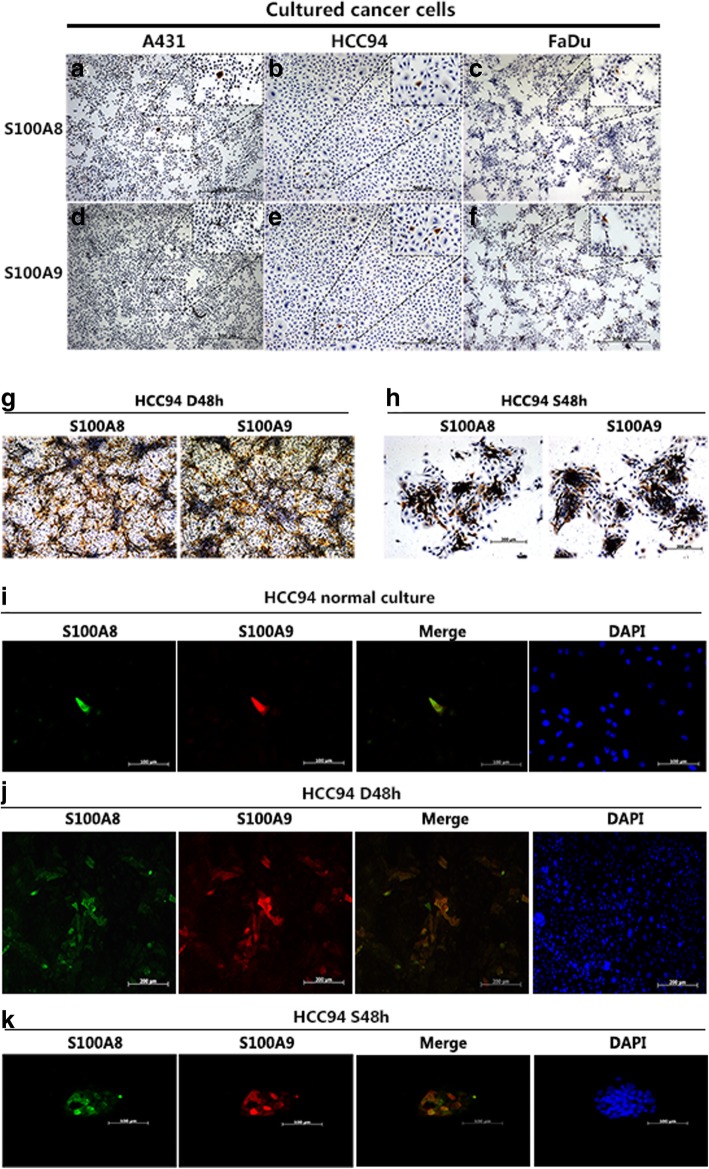
Immunohistochemical analysis of S100A8/S100A9 protein expression in SCC cells. The staining of S100A8/S100A9 in A431 cells (**a** and **d**), HCC94 cells (**b** and **e**) and FaDu cells (**c** and **f**) in normal culture conditions. Cells were cultured in dense condition (D48h) and suspension condition (S48 h) for 2 days, the staining of S100A8/S100A9 in HCC94 cells were analyzed by immunohistochemical (**g** and **h**). Double immunofluorescence staining of S100A8/S100A9 in normal, dense and suspension culture condition, as well as DAPI staining in HCC94 cells (**i-k**)

Recent studies have shown that S100A8/S100A9 is associated with various neoplastic disorders [31–33]. However, the regulation of both expressions in SCCs is not well defined. Therefore, we examined the expression of S100A8/S100A9 in squamous cell carcinoma cell lines A431, HCC94 and FaDu. Interestingly, we found that less than 1% S100A8/S100A9-positive cells were detected in all tested cell lines under normal culture conditions (Fig. 1a-f). It has reported that S100A9 expression can be induced in normal primary keratinocytes (HEKn) under dense culture [34]. Therefore, to investigate whether S100A8/S100A9 could also be induced under dense conditions, HCC94 cells were cultured at high densities (‘dense’, 100,000 cells/cm^2^). Immunohistochemical staining revealed that S100A8/S100A9-positive cells could be induced by dense treatment compared with low cell density (hereafter called ‘sparse’, 10,000 cells/cm^2^). The percentage of S100A8/S100A9-positive cells increased from less than 1% under normal culture conditions to above 40% after dense 2 days (Fig. 1g) and then, significantly decreased after the dense cells were reseeded at the pre-dense density and returned to the original ratio after three cell passages (Additional file 2: Figure S1). Suspension culture is another inducer of S100A9 expression in keratinocytes [34]. To explore whether S100A8/S100A9 may also induced by suspension in SCC cells, the HCC94 cells in suspension reattached to a slide for 12 h and then examined by immunohistochemistry. The expression of S100A8/S100A9 significantly up regulated in suspension culture (Fig. 1h). Subsequently, double immunofluorescence staining further confirmed that S100A8/S100A9-positive cells co-localized in all culture conditions (Fig. 1i-k). Collectively, these results indicate that cell density and cell morphology can regulate S100A8/S100A9 co-expression and both protein display co-localization in SCC cells.

### S100A8/S100A9-positive cells are co-inducible in vivo

**Fig. 2:**
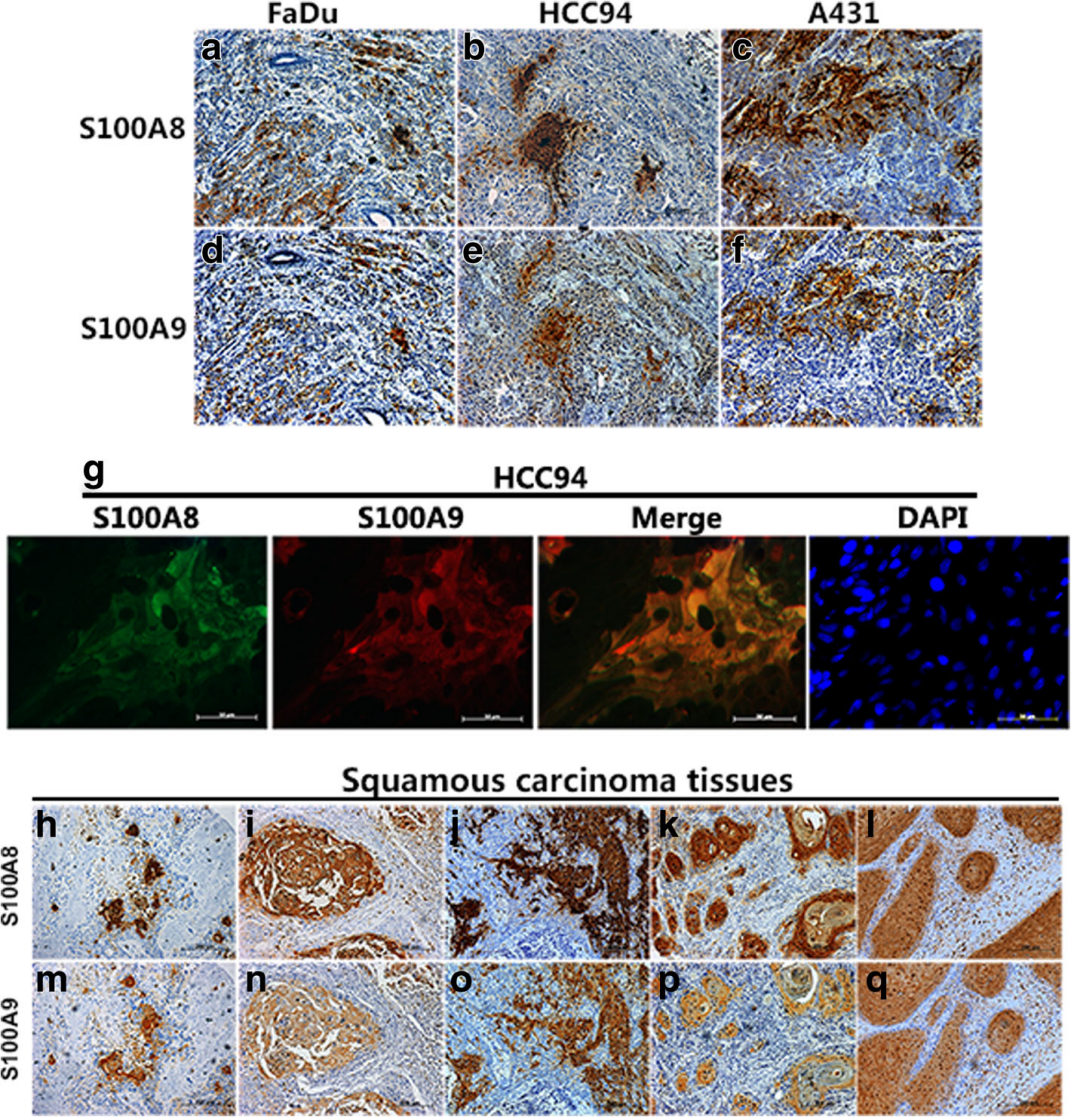
Co-induction and co-localization of S100A8/S100A9 expression in vivo. The staining of S100A8/S100A9 was examined in xenografts derived from FaDu (**a** and **d**), HCC94 (**b** and **e**), and A-431 cells (**c** and **f**) by immunohistochemistry in two consecutive sections. Co-localization of S100A8/S100A9 expression further confirmed by double immunofluorescence with DAPI staining in HCC94 xenograft (**g**). The staining of S100A8/S100A9 in the lung (**h** and **m**); esophageal (**i** and **n**); cervical (**j** and **o**); oral (**k** and **p**); and skin SCC specimens (**l** and **q**) were examined by immunohistochemistry in two consecutive sections

Based on the above results, we hypothesized that S100A8/S100A9 may also be induced and co-expressed or co-localized in vivo. To test our guess, we examined S100A8/S100A9 expression pattern in xenografts derived from FaDu, A431, and HCC94 cells by immunohistochemistry in two consecutive sections. As expected, the percentage of S100A8/S100A9-positive cells was raised from less than 1% in FaDu, HCC94 and A431 cells in vitro to score 1, score 2 and score 3 (IHC staining score: 0, no positive cells; 1, < 10% positive cells; 2, > 10 and < 50% positive cells; 3, > 50 and < 75% positive cells; 4, > 75% positive cells) in their corresponding xenografts, respectively (Fig. 2a-f). Importantly, co-expression of S100A8/S100A9 also detected in all tested xenografts by immunohistochemistry, and the co-localization further confirmed in HCC94 xenograft by double immunofluorescence analysis (Fig. 2g). These results directly support the hypothesis that S100A8/S100A9 co-induction in SCC cells also occurs in vivo and implies that the functions of S100A8/S100A9 in SCC may be dependent on each other.

To investigate the expression pattern of S100A8/S100A9 in different types of SCCs, five types of SCC tissue arrays with 257 SCC cases examined by immunohistochemistry in two consecutive sections. S100A8/S100A9 expression could be detected in 74.4% of lung SCCs, 100% of esophageal SCCs, 100% of cervical SCCs, 96% of oral SCCs, and 95% of skin SCCs stained positive for both S100A8/S100A9 (Additional file 1: Table S3). Although S100A8/S100A9 expression was frequently focal positive immunostaining in the well or moderately differentiated areas even with in one specimen, but both proteins showed the similar staining distributions (Fig. 2h-q). These results indicate that the co-expression of S100A8/S100A9 may be a common feature of SCCs, at least in well and moderately tissues and /or regions.

### S100A8/S100A9 co-induction is regulated by Hippo-YAP pathway

**Fig. 3:**
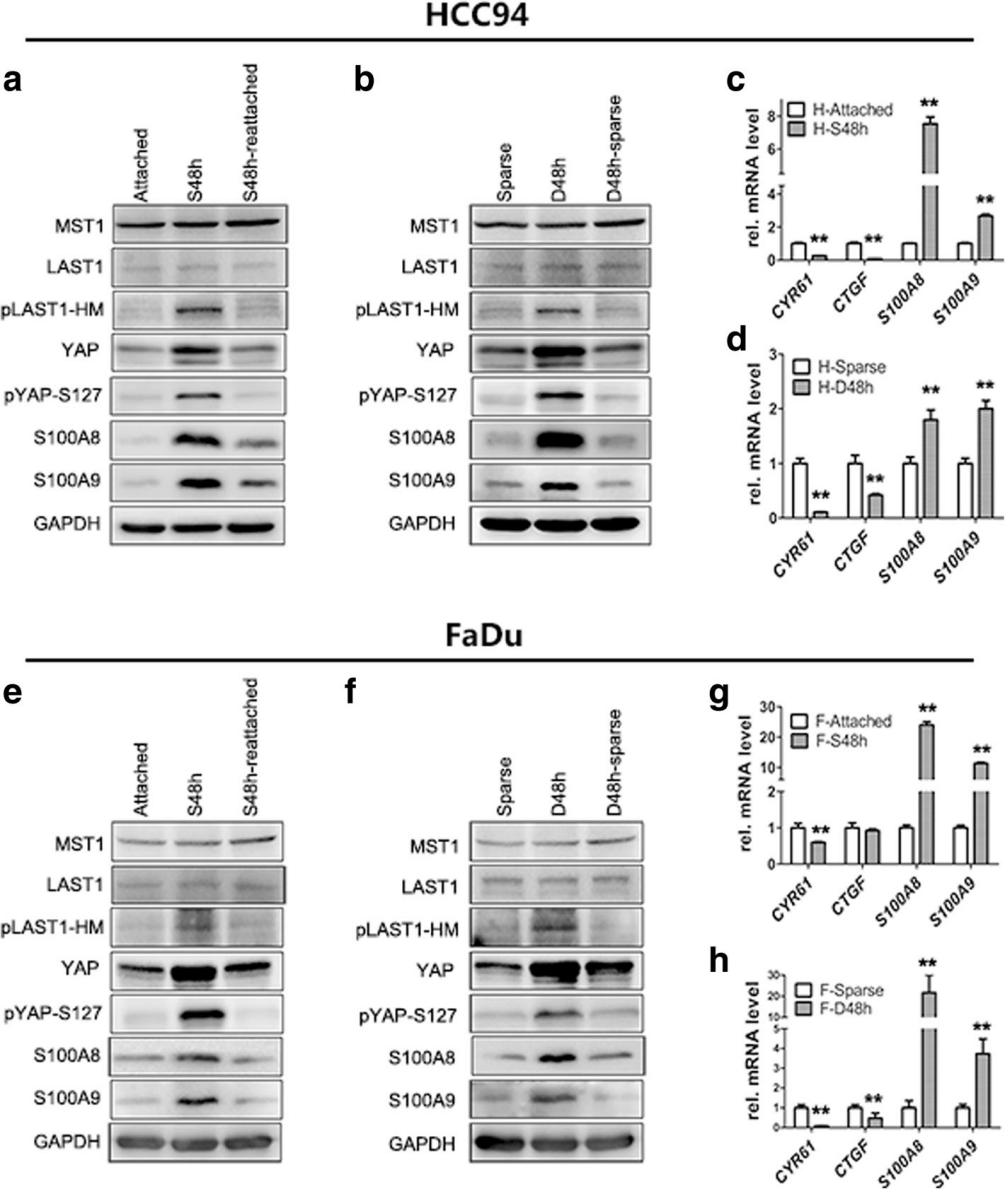
S100A8/S100A9 induction accompanied by inactivation of YAP and activation of Hippo pathway. Western blot analyses of S100A8/S100A9 and Hippo pathway in HCC94 (**a** and **b**) and FaDu (**e** and **f**) cells. Cells cultured in suspension for 2 days (S48 h) and then reattachment for 1 day (S48 h-reattached). Cells cultured densely for 2 days (D48h) and then relief from dense culture (D48h-sparse). GAPDH was used as a loading control. The expression of *S100A8*, *S100A9*, *CTGF* and *CYR61* were analyzed by qPCR in HCC94 cells (**c** and **d**) and FaDu cells (**g** and **h**). Error bar, SD of three different experiments. *p < 0.05, **p < 0.01; t-test

**Fig. 4:**
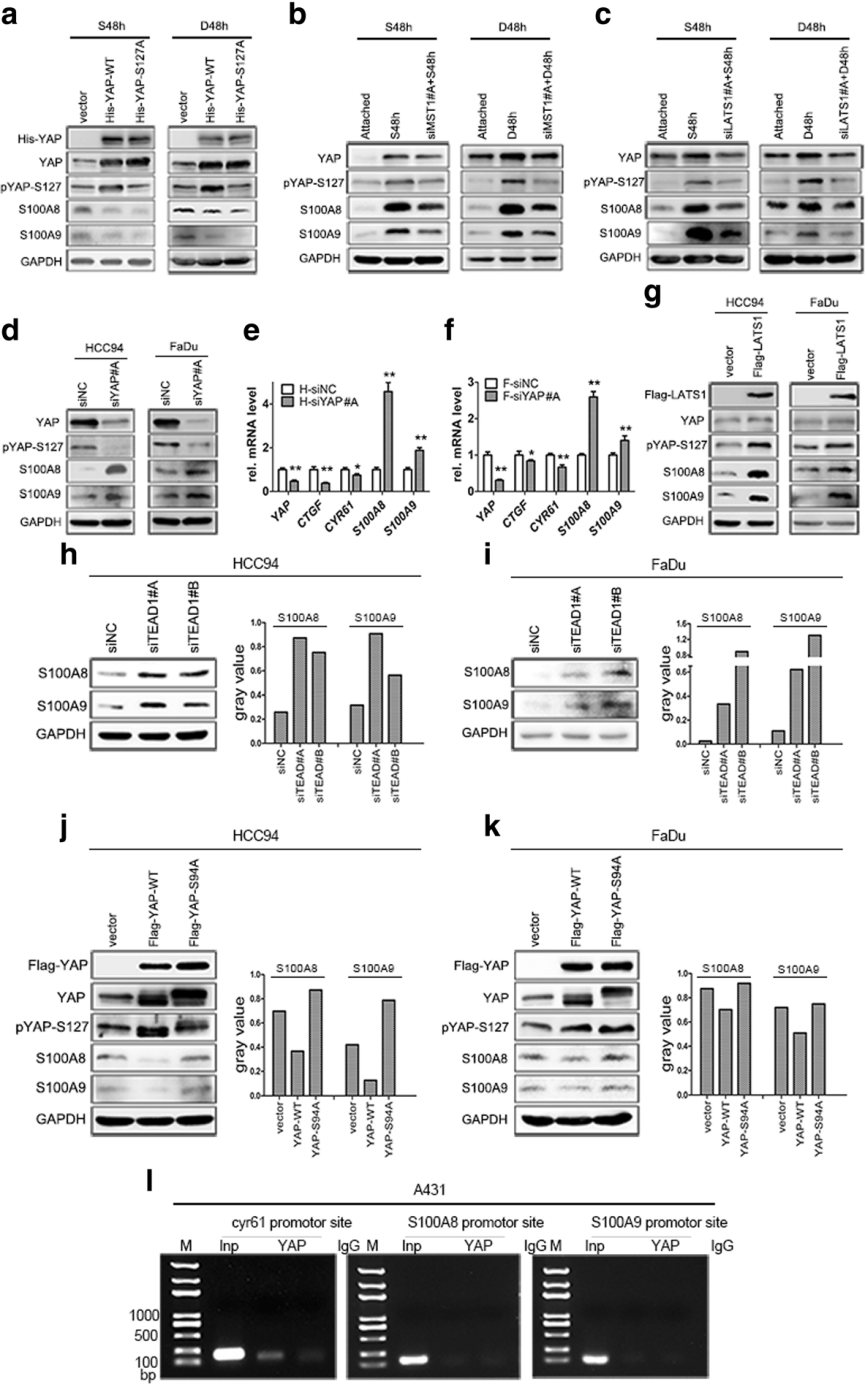
The Hippo pathway is responsible for S100A8/S100A9 induction. HCC94 cells were transfected with His-YAP-WT and His-YAP-S127A under suspension and dense culture condition (**a**). MST1 and LATS1 were knockdown by corresponding siRNAs in suspension and dense cultured HCC94 cells (**b** and **c**). Deletion of YAP in normal attached HCC94 and FaDu cells, the expression of S100A8/S100A9 was tested by western blot (**d**), and *CTGF*, *CYR61* were detected by qPCR (**e** and **f**). Overexpression of LATS1 in normal attached HCC94 cells and FaDu cells (**g**), anti-flag tag antibody was used to judge the transfection efficiency. TEAD1 was deleted by two specific siRNAs in HCC94 (**h**) and FaDu (**i**) cells, the expression of S100A8/S100A9 was detected by western blot and the gray value of S100A8/S100A9 was analyzed by ImageJ Launcher. HCC94 cells (**j**) and FaDu cells (**k**) were transfected with Flag-YAP-WT and Flag-YAP-S94A plamids, anti-flag tag antibody was used to judge the transfection efficiency. YAP was not binding on S100A8/S100A9 promoter sites were detected by CHIP analysis using anti-YAP versus IgG control antibody, CYR61 was as positive control (**l**)

Both suspension and dense can activate the Hippo pathway via actin cytoskeleton reorganization [26, 35]. Based on the above results, we hypothesized that the Hippo/YAP pathway may participate in S100A8/S100A9 induction. As expected, first, we found the positive correlation of S100A8/S100A9 induction and the Hippo pathway activation. In the suspended and dense cells, the expression of S100A8/S100A9 in mRNA and protein levels was significantly increased while the Hippo pathway was activated, as indicated by an increase in the status of LATS-HM (LATS1-T1079) and YAP (YAP-S127) phosphorylation (Fig. 3a, b, e, f; Additional file 2: Figure S2a-c), as well as transcription suppression of *CTGF* and *CYR61*, two direct endogenous markers of YAP activity (Fig. 3c, d, g, h). Next, the decrease of S100A8/S100A9 co-expression also accompanied by inhibition of the Hippo pathway after recovery of cell attachment or relief from dense culture. These results suggest the Hippo-YAP pathway may regulate S100A8/S100A9 expression in SCC cells. To test this hypothesis, YAP-S127A (a constitutively activated form of YAP) or YAP-WT plasmid was transfected into HCC94 cells and then cultured these cells under suspension and dense conditions. As expected, overexpression of YAP-S127A led to the more obvious suppression of S100A8/S100A9 than YAP-WT overexpression (Fig. 4a). Similarly, inactivation of the Hippo pathway by knockdown of MST1 or LATS1 dramatically decreased S100A8/S100A9 expression as well as YAP phosphorylation in suspended or dense HCC94 cells (Fig. 4b, c; Additional file 2: Figure S2d). The knockdown efficiency of two different specific LATS1 siRNAs and MST1 siRNAs detected by qPCR (Additional file 2: Figure S3a, b). Conversely, depletion of YAP induced S100A8/S100A9 co-expression in HCC94, FaDu and A431 cells (Fig. 4d-f; Additional file 2: Figure S2e, f), the knockdown efficiency of two different specific YAP siRNAs were detected by qPCR (Additional file 2: Figure S3c). This inducible phenomenon of S100A8/S100A9 also detected in overexpression of LATS in HCC94 and FaDu cells (Fig. 4g; Additional file 2: Figure S2g). These results confirm that the Hippo-YAP pathway can regulate S100A8/S100A9 expression.

Since TEAD is indispensable for YAP to regulate transcription as a co-activator or corepressor in the nucleus [36–38], we first transiently knocked down TEADs in attached HCC94 and FaDu cells using two specific TEADs siRNAs. Interestingly, only silencing of TEAD1 led to a substantial increase of S100A8 and S100A9 expression (Fig. 4h, i; Additional file 2: Figure S2h, i), the knockdown efficiency of two different specific TEAD1 siRNAs were detected by qPCR (Additional file 2: Figure S3d). However, knockdown of the other three TEADs (TEAD2, TEAD3, TEAD4) almost had not any effects (data not shown). YAP-S94 is essential for the combination of YAP and TEAD [26, 38], overexpression of YAP-S94A (a mutation of YAP 94 site) marginally affected S100A8/S100A9 co-expression compared with YAP-WT (Fig. 4j, k). However, the direct association of YAP with the promoter of S100A8/S100A9 was not observed by chromatin immunoprecipitation (ChIP) (Fig. 4l), supporting that the transcription of S100A8/S100A9 may be indirectly regulated by YAP/TEAD1 complex.

To test whether the Hippo pathway activated in vivo, we also examined YAP and pYAP-S127 expression pattern in xenografts derived from A431 cells by immunohistochemistry in two consecutive sections. Expectedly, the low expression of YAP and the high expression of pYAP-S127 were detected in the same area, indicating that the Hippo pathway was indeed activated in xenografts. These results suggest that the induction of S100A8/A9 expression in vivo also related to the Hippo pathway (Additional file 2: Figure S4). Collectively, these data unequivocally demonstrate for the first time that activation of the Hippo pathway is a critical step for S100A8/S100A9 induced by cell shape and cell density in SCC cells.

### F-actin disruption enhances S100A8/S100A9 co-expression via activation of the Hippo pathway

**Fig. 5:**
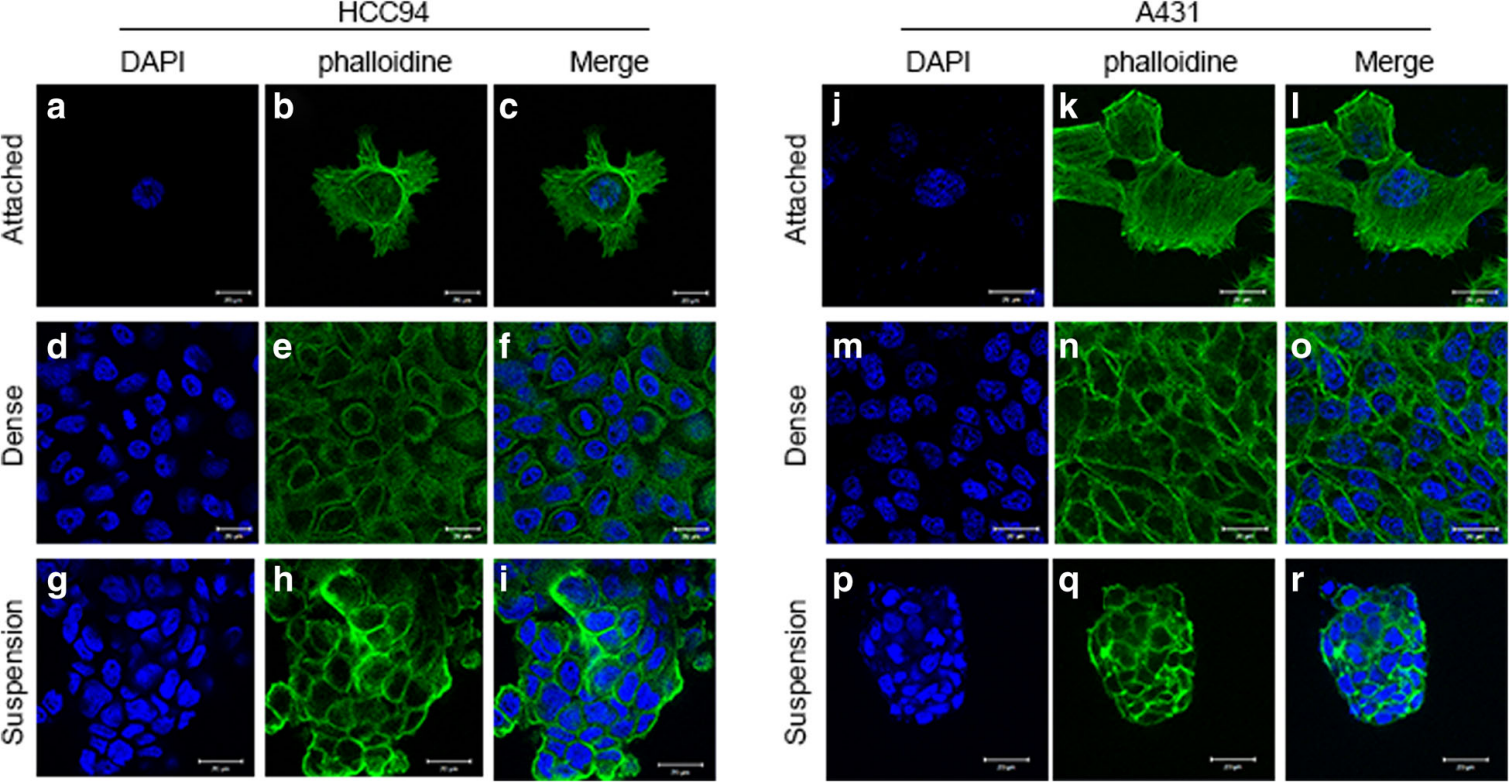
F-actin distribution in normal, dense, suspension cell cultures. At low cell densities, the F-actin in stress fibers is thick and abundant in HCC94 (**a-c**) and A431 (**j-l**) cells. At high cell densities, stress fibers were thin and less evident in HCC94 (**d-f**) and A431 (**m-o**) cells. No stress fiber were observed in suspension cultured HCC94 (**g-i**) and A431 (**p-r**) cells

It has reported that suspension and dense culture activate the Hippo pathway by F-actin cytoskeleton reorganization [26, 35]. Thus, we examined the intracellular F-actin distribution in normal, suspension and dense cell cultures. At low cell density, F-actin bundles exist to supporting cell morphology (Fig. 5a-c, j-l). However, bundles formed by F-actin was not observed in the suspension and dense cultured cells, F-actin tended to depolymerize and depolymerized F-actin aggregated around the cell membrane (Fig. 5d-i, m-r).

**Fig. 6:**
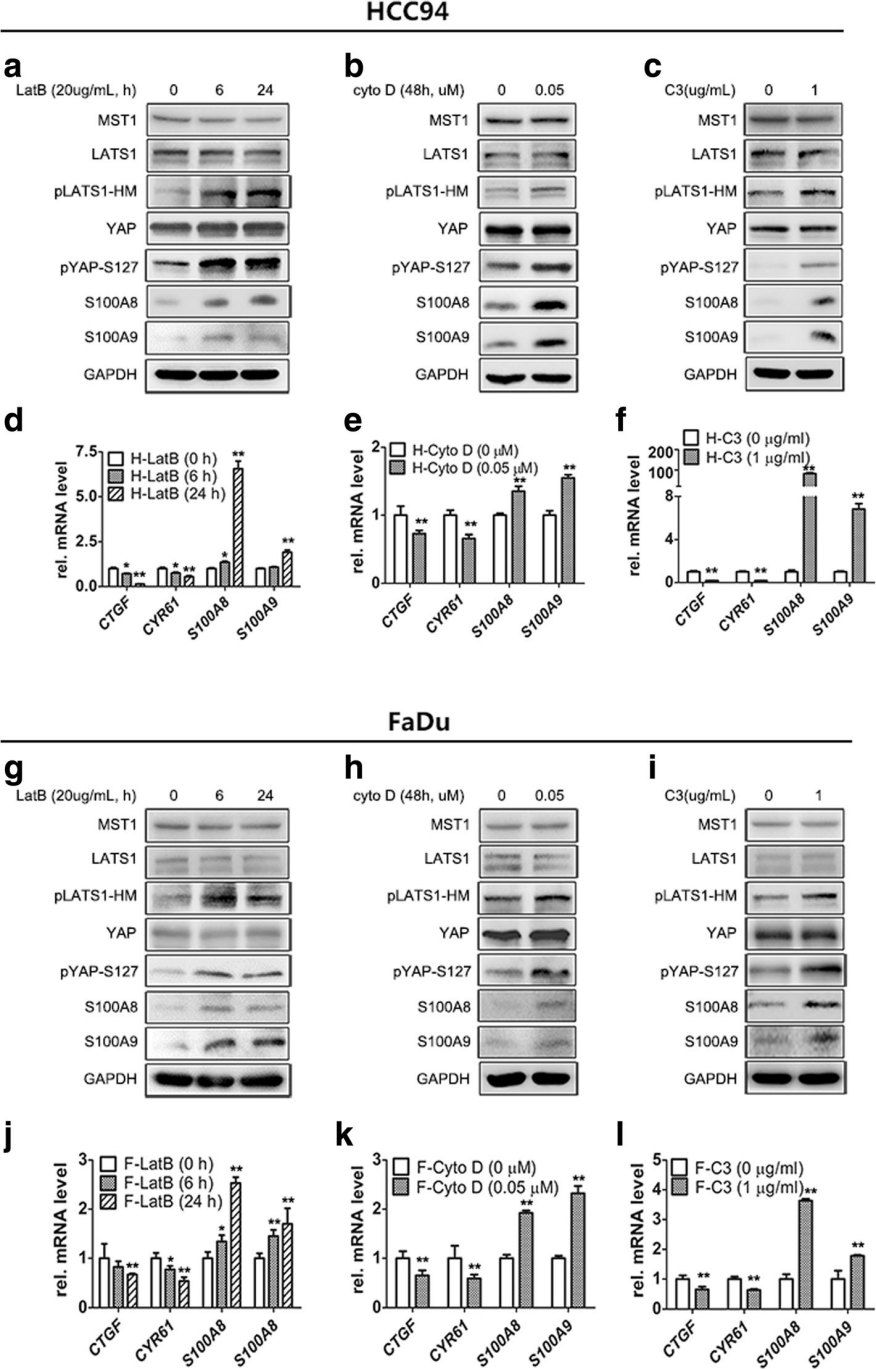
S100A8/S100A9 induction mediated by actin cytoskeleton via the Hippo pathway. HCC94 (**a**) and FaDu (**g**) cells were treated with LatB (20μg/mL) for 6 h and 24 h. HCC94 (**b**) and FaDu (**h**) cells were cultured for 48 h in the present of CytoD (0.05, 0.1 uM). C3 (1 μg/mL) was added to HCC94 cells (**c**) and FaDu cells (**i**) with serum-free growth medium for 4 h prior to harvesting for Western blotting analyses. *CYR61*, *CTGF*, *S100A8*/*S100A9* was detected by qPCR in HCC94 (**d-f**) and FaDu (**j-l**) cells

If F-actin is also involved in YAP-mediated S100A8/S100A9 induction, disruption of F-actin should affect the expression of S100A8/S100A9. To test this hypothesis, we treated the HCC94 and FaDu cells with two drugs, LatB and cytoD, which could disrupt the F-actin cytoskeleton by preventing actin polymerization and capping filament plus ends respectively [26]. As expected, treatment of cells with LatB for 24 h resulted in F-actin depolymerized (Additional file 2: Figure S5d-f), and western blot showed that S100A8/S100A9 was significantly induced and accompanied by the Hippo pathway activation (Fig. 6a, b, g, h) which was indicated by a significant decrease of *CYR61* and *CTGF* mRNA level (Fig. 6d, e, j, k). Similar results were also observed in the same cells after treatment with Rho inhibitor C3 that could induce the dephosphorylation of YAP by regulating actin cytoskeleton [32] (Fig. 6c, f, i, l, Additional file 2: Figure S5g-i). Together, our findings indicate that actin cytoskeleton remodeling either by cells suspension and dense culture or disruption of F-actin induces S100A8/S100A9 co-expression through activation of the Hippo pathway.

### S100A8/S100A9 promotes cell proliferation and inhibits squamous differentiation

**Fig. 7:**
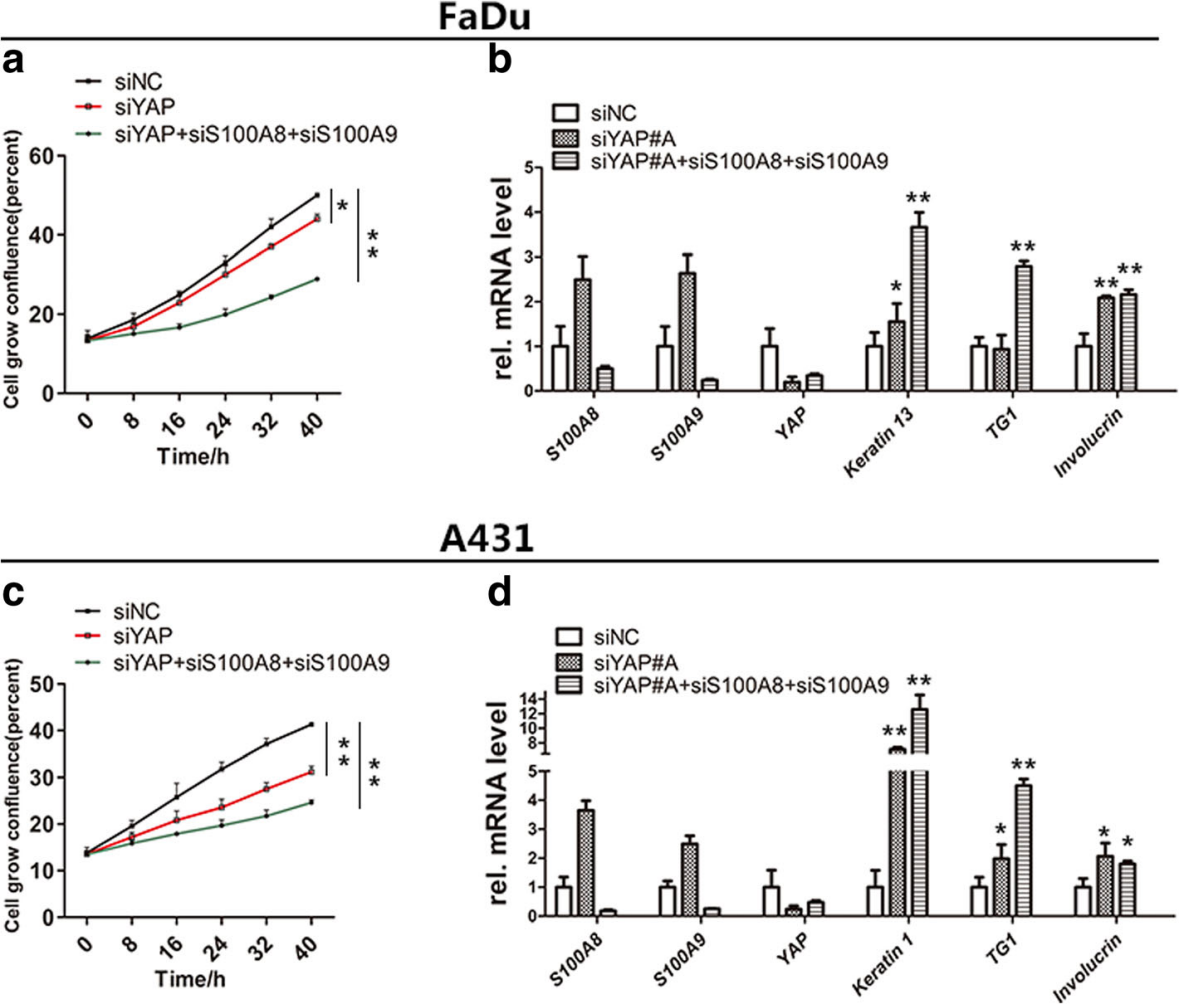
Loss of S100A8/S100A9 and YAP leads to cell differentiation and growth inhibitions. The cell proliferation of single deletion of YAP, S100A8/S100A9 or both in FaDu cells (**a**) and A431 cells (**c**) were determined by IncuCyte ZOOM long time live cell image monitoring system. Differentiation genes were induced by delete YAP, S100A8/S100A9 in FaDu cells (**b**) and A431 cells (**d**). Error bar, SD of three different experiments. *p < 0.05, **p < 0.01; *t*-test

It has reported that S100A8/S100A9 forms heterodimers and plays an important role in regulating cells proliferation and apoptosis in some normal and cancer cells [32, 39–43]. To explore the effect of S100A8/S100A9 on SCC cells, we performed depletion of YAP with or without combined with knockdown S100A8/S100A9 in A431 and FaDu cells. The results revealed that depletion of YAP and S100A8/S100A9 together resulted in the more obvious suppression of cells proliferation and promotion of squamous differentiation compared with silencing of YAP alone, as indicated by an increase of squamous differentiation markers including *Keratin 1*, *Keratin 13* and *TG1*, as well as *involucrin* [34, 37, 44, 45] (Fig. 7a-d). These results suggest that S100A8/S100A9 and YAP both play the similar role in promoting cell proliferation and inhibiting squamous differentiation. In these cases, the function of YAP seems more effective than S100A8/S100A9, becauseS100A8/S100A9 induced by YAP knockdown was not enough to counteract the effect of YAP silencing on cell proliferation and differentiation. Collectively, our findings indicate that S100A8/S100A9 and YAP function as the positive regulator of cell proliferation and negative regulator of cell differentiation in SCC cells.

### S100A8/S100A9 inhibits cell apoptosis induced by suspension and dense culture

**Fig. 8:**
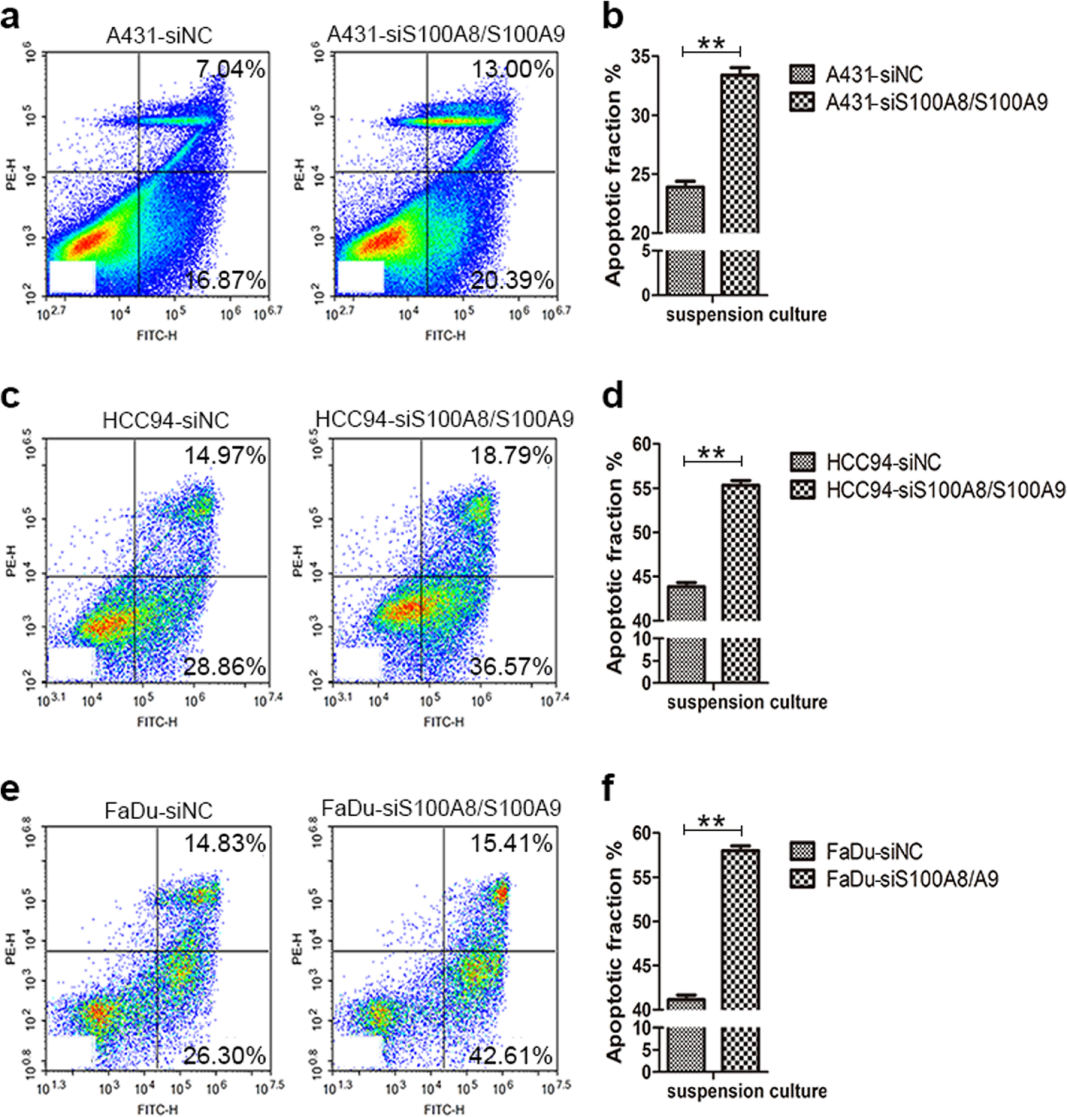
S100A8/S100A9 inhibit cell apoptosis induced by suspended culture. A431 cells (**a** and **b**), HCC94 cells (**c** and **d**) and FaDu cells (**e** and **f**) were transfected with S100A8/S100A9 specific siRNAs, 24 h later cells were suspended culture 48 h. The proportion of cell apoptosis was measured by Flow cytometry

**Fig. 9:**
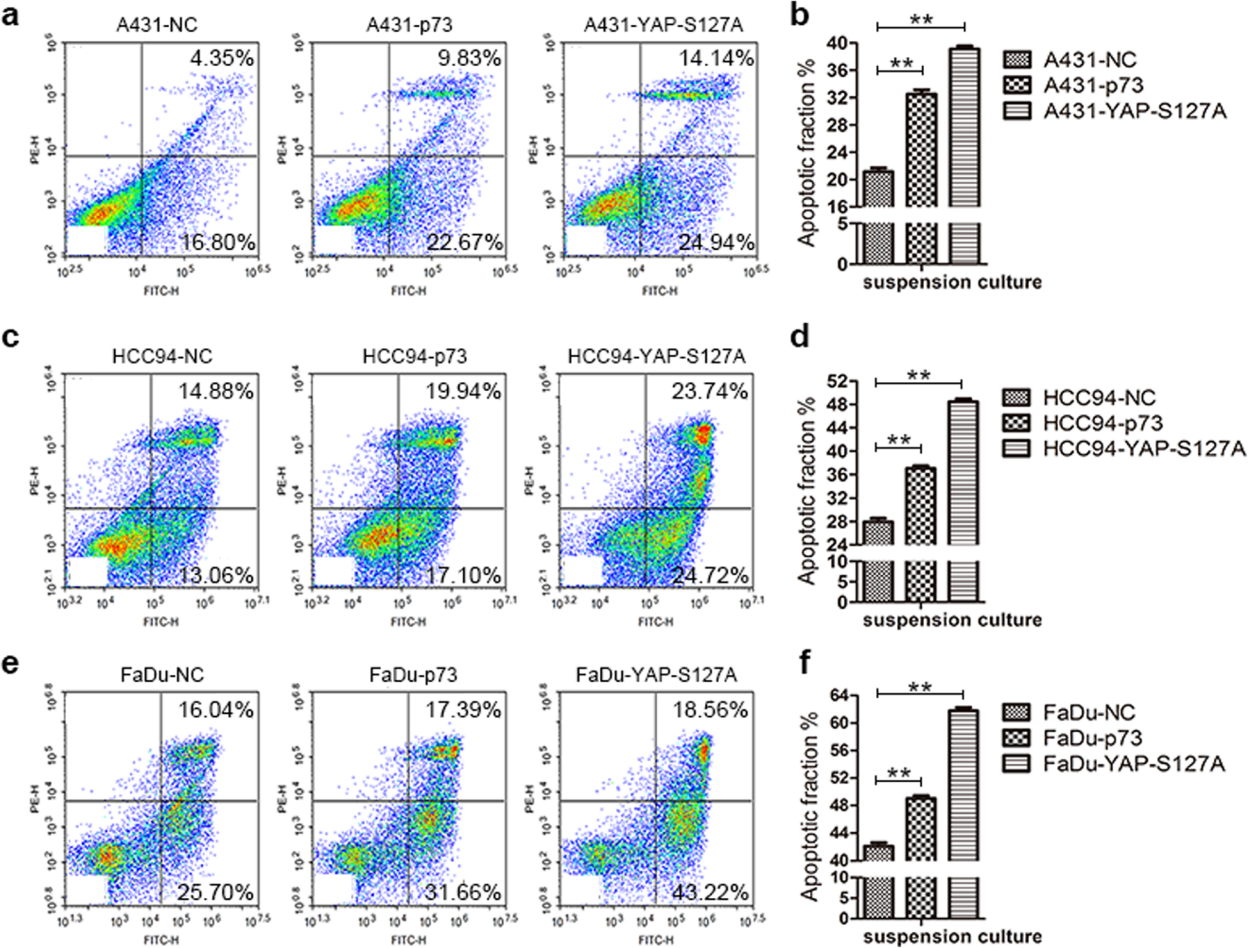
YAP and p73 promote cell apoptosis induced by suspended culture. A431 cells (**a** and **b**), HCC94 cells (**c** and **d)** and FaDu cells (**e** and **f**) were transfected with p73 and YAP-S127A plasmids, 24 h later cells were suspended culture 48 h. The proportion of cell apoptosis was measured by Flow cytometry

Because suspension and dense culture leads to cell apoptosis, even for cancer cells, we next explore the function of S100A8/S100A9 in suspended and dense SCC cells. The results showed that silencing of S100A8/S100A9 together significantly promoted cell apoptosis in suspended and dense cells compared with the control groups (Fig. 8, and Additional file 2: Figure S6). It has been reported that YAP can bind to p73 during cells are cultured in an apoptosis-inducing environment, such as suspension, and the complex of YAP/p73 initiates the expression of apoptosis-related genes [46, 47]. To investigate whether YAP and p73 participate in SCC cell apoptosis, we introduced YAP-S127 and p73 plasmids into three kinds of SCC cells, and then cultured these cells in suspension and high density for 48 h. The results showed that overexpression of YAP-S127 and p73 significantly increased the proportion of apoptosis in all tested cells relative to the control groups (Fig. 9, Additional file 2: Figure S7). These suggest that YAP may play dual functions in SCC cells depending on the cells cultured microenvironment. Therefore, for suspended and high-density cultured cells, inactivation of YAP and induced expression of S100A8/S100A9 are beneficial to cell survival.

## Discussion

In the present study, we examined the expression of S100A8/S100A9 in three SCC cells, less than 1% of S100A8/S100A9-positive cells observed in all the tested cells under the normal culture condition. However, we observed interesting phenomena. When cells cultured in dense, the percentage of S100A8/S100A9-positive cells were obviously increased and co-localized but rapidly returned to the original ratio after recovery of low cell density culture. Similar phenomena were also detected in suspend cells. Importantly, S100A8/S100A9 mRNA and protein levels were always consistent with the percentage of their positive cells. These results suggest that S100A8/S100A9 is inducible in vitro, which depends on cell shape and cell density. Next, we also examined the expression pattern of S100A8/S100A9 in vivo. We found that the expression characteristics of S100A8/S100A9 were marked heterogeneity and display co-expression and co-localization in three SCC cell lines’ corresponding xenografts. Interestingly, when we compared the percentage of S100A8/S100A9 immunostaining in cultured SCC cells and their corresponding xenograftes, we unexpectedly found that there were an inconsistent proportion of S100A8/S100A9-positive cells in vitro and in vivo. Although less than 1% of S100A8/S100A9-positive cells were observed in cultured SCC cells, a greater number of both positive cells were detected and displayed co-localization in the all tested xenografts. These results indicate that S100A8/S100A9 can also be co-induced in vivo. The co-expression of S100A8/S100A9 was also observed in 257 SCC specimens derived from SCC of lung, esophagus, cervix, oral cavity, and skin by immunochemistry and clinical specimen. Taken together, our results indicate that S100A8/S100A9-negative cells can convert into the positive cells and increase their expression levels either by suspension (change of cell morphology) or by dense culture (altering cell density), vice versa. Based on the above results, we have reason to speculate that the inter-conversion of S100A8/S100A9-negative and -positive cells also happen in vivo.

Subsequently, we found and confirmed that cell shape and cell density controlled S100A8/S100A9 co-expression through F-actin-mediated the Hippo pathway. The following results supported our conclusion; first, we overexpressed LATS1 or depleted the endogenous YAP in order to activation of the Hippo pathway in SCC cells. The results revealed that S100A8/S100A9 co-expression was significantly increased. Conversely, inactivation of the Hippo pathway by either deletion of LATS1 and MST1 or overexpression of YAP-WT and YAP-S127A markedly blocked suspension- and dense-induced S100A8/S100A9 co-expression. Second, we demonstrated that the induction of S100A8/S100A9 and activation of the Hippo pathway were also detected in attached cells after disruption of F-actin by LatB, CytoD and C3. Finally, we proved that TEAD1 was a cofactor of YAP for repressing S100A8/S100A9 co-expression, which further confirmed by the transfection of YAP-S94A into the cells. Although it has been reported that YAP can be combined with numerous transcription factors, including ErbB4, Runx2, and TEAD, but TAED is one of the most important ones [38, 39, 48–50]. Therefore, we do not rule out the possibility of other transcription factors to regulate S100A8/S100A9 expression by interacting with YAP in SCC cells. Interestingly, it has reported that YAP-TEAD1 complex paly dual functions of activation and inhibition of gene transcription via recruitment of different complex to target gene [50–52]. Our study provides the first biological evidence that S100A8/S100A9 expression is really, but indirectly regulated by the Hippo/YAP pathway in SCC cells. These results suggest that there is another intermediate protein to mediate the regulation of S100A8/S100A9 expression by YAP-TEAD1 complex.

Except of the actin cytoskeleton, the microtubule cytoskeleton is also reorganized during cell detachment, which in turn activating the Hippo/YAP pathway. Interestingly, only detachment-induced YAP phosphorylation can be strongly blocked by nocodazole, but not attachment-induced YAP dephosphorylation [38]. In addition, other signal pathways can also regulate the activation of the Hippo-YAP pathway, such as G-protein coupled receptor and E-cadherin-catenin complex [53, 54], Therefore, it is not impossible that other pathways may also participate in regulating of S100A8 and S100A9 expression except of the actin cytoskeleton .

Although the aberrant expressions of S100A8/S100A9 has reported in a variety of cancer tissues, the functional studies are also necessary to pay more attention. It has reported that, in SCC12 cells (cutaneous SCC cell line), overexpression of S100A8 and/or S100A9 increase cells proliferation and migration capacity in vitro, as well as promote tumors growth in vivo [31]. In the present study, we demonstrated that deletion of YAP combined with or without knockdown of S100A8/S100A9 inhibited cells proliferation but promoted squamous differentiation, but triple deletion have a better effect. These results support that although YAP activity and S100A8/S100A9 expression display the negative correlation in the test SCC cells, they both perform the same and similar effects on cell proliferation and differentiation in the tested SCC cells. Thus, we speculate that YAP and S100A8/S100A9 might act as the compensatory function depending on cell microenvironment. In normal adherent cultured cells, the Hippo pathway is in a closed state. YAP binding with TEAD in the nucleus, play a role in promoting cell proliferation and inhibiting of cell differentiation. YAP downstream protein ‘X’ binds to the promoter of S100A8/S100A9, inhibiting S100A8/S100A9 expression. When SCC cells are detached or cultured in high density, the Hippo pathway are activated and nuclear YAP are decreased so that S100A8/S100A9 lost the inhibitory effect on protein ‘X’, which leads to them induction (Additional file 2: Figure S8). The induced S100A8/S100A9 instead of YAP plays the similar effect on promoting cell proliferation and inhibiting differentiation. Although we did not find out protein ‘X’ in the present study, this work is ongoing in our laboratory. More importantly, we found that under suspension and dense culture YAP and S100A8/S100A9 play the opposite biological function, YAP and p73 promoted cell apoptosis and S100A8/S100A9 inhibit apoptosis. Therefore, we hypothesized that the inactivation of YAP and the induction of S100A8/S100A9 in the above-mentioned conditions may be a mechanism for cancer cells resisting apoptosis and maintaining survival. In the process of metastasis through blood and lymph, cancer cells are suspended due to lack of matrix adhesion. To overcome anoikis, cancer cells develop a series of coping strategies, such as delaying the time of anoikis by autophagy and mutual phagocytosis, and improving the survival rate of metastatic cells [55]. Our results also revealed that the inactivation of YAP and the induction of S100A8/S100A9 could significantly increase the survival of cancer cells, which may also be a mechanism by which cancer cells overcome anoikis during metastasis.

## Conclusion

This study uncovers for the first time that S100A8/S100A9 is inducible both in vitro and in vivo, and both proteins display co-expression and /or co-localization. Actin cytoskeleton reorganization plays a critical role in control of S100A8/S100A9 co-expression through the Hippo-YAP pathway. Induced S100A8/S100A9 promoted cell proliferation, inhibit cell differentiation and apoptosis.

## Additional files

Additional file 1: **Methods**. **Table S1.** siRNA sequences. **Table S2.** Primers used for qPCR. **Table S3** S100A8 and S100A9 expression in SCC tissues. (DOCX 25 kb)

Additional file 2: **Figure S1.** The Expression of S100A8 and S100A9 in HCC94 cells. **Figure S2.** Induction of S100A8 and S100A9 expression in A431 cells. **Figure S3.** Silencing effect of siRNAs in A431 cells. **Figure S4.** The expression of YAP and pYAP-S127 in xenografts. **Figure S5.** S100A8/A9 inhibit cell apoptosis induced by dense culture. **Figure S6.** YAP and p73 promote cell apoptosis induced by dense culture. **Figure S7.** Diagram to summarize S100A8 and S100A9 induction procedure. **Figure S8.** Diagram to summarize S100A8 and S100A9 induction procedure. **(a)** In normal adherent cultured cells, the Hippo pathway is in a closed state. YAP binding with TEAD in the nucleus, play a role in promoting cell proliferation and inhibiting of cell differentiation. YAP downstream protein ‘X’ binds to the promoter of S100A8 and S100A9, inhibiting S100A8 and S100A9 expression. **(b)** When SCC cells are detached or cultured in high density, the Hippo pathway are activated and nuclear YAP are decreased so that S100A8 and S100A9 lost the inhibitory effect on protein ‘X’, which leads to them induction. (DOCX 3844 kb)

## Data Availability

All data in our study are available upon request.

## Abbreviations

AA: Arachidonic acid
C3: Botulinum toxin C3
ChIP: Chromatin immunoprecipitation
Cyto D: Cytochalasin D
DAPI: 4′, 6-diamidino-2-phenylindole
FITC: Fluorescein isothicocyanate
LatB: Latrunculin B
SCC: Squamous cell carcinomas
TRTIC: Tetramethyl thodamine isothiocyanate
