# Supplementary material for: The expression of S100A8/S100A9 is inducible and regulated by the Hippo/YAP pathway in squamous cell carcinomas
Source: BMC Cancer. 2019 Jun 17;19:597. doi: 10.1186/s12885-019-5784-0 (PMC6580480; doi:10.1186/s12885-019-5784-0)
Supplement: Supplementary file 1 — Methods. Table S1. siRNA sequences. Table S2. Primers used for qPCR. Table S3 S100A8 and S100A9 expression in SCC tissues. (DOCX 25 kb) [file 12885_2019_5784_MOESM1_ESM.docx]

**Methods**

**Plasmids and Reagents**

The pcDNA4-His-YAP WT; S127A and pCMV14-Flag-YAP WT; S94A vectors were kindly provided by Dr. Zhang (Mayo Clinic College of Medicine, USA). For pCMV14-Flag-LATS1, the LATS1 (NCBI Gene ID: 9113) cDNA fragment was amplified using 5’-CGGGGTACCATGAAGAGGAGTGAAAAG-3’ and 5’-GCTCTAGAAACATATACTAGATCGCGATTT-3’, and then was cloned into the mammalian expression vector pCMV14 (Invitrogen, Carlsbad, CA, USA) using *KpnI* and *XbaI* restriction enzymes (Takara, Dalian, China). Latrunculin B (L5288) and Cytochalasin D (C8273) were purchased from Sigma (Sigma-Aldrich Ltd, Dorset, UK). Botulinum toxin C3 (CT04) was purchased from Cytoskeleton (Denver, USA).

**siRNA and transfection**

To silence the expression of YAP, LATS1, MST1, TEAD1, S100A8 and S100A9, all siRNAs as well as the non-targeting control siRNA were purchased from Gene Pharma (Shanghai, China) and transfected using the Transfection Reagent (Polyplus, NY, USA) according to the manufacturer's protocol (Additional file 1: Table S1).

**RT-PCR**

Total RNA was extracted from cells for the generation of single-stranded cDNA. Quantitative RT-PCR (qPCR) was performed using an ABI 7300 Real-time PCR System (Life Technologies Ltd, Paisley, UK) with the Power SYBR® Green PCR Master Mix (Life Technologies Ltd) in a final volume of 20 μL. GAPDH was used as an endogenous control for each sample. The primers used for each of the genes are listed (Additional file 1: Table S2).

**Immunofluorescence staining**

For suspended cells, cells were cultured in suspension for 24 hours and reattached to a slide for 12 h. Cells were fixed with 3.4% paraformaldehyde for 20 min and then permeabilized with 0.5% PBS-Triton X-100 (Ding guo, Beijing, China). After blocking in 3% PBS-BSA for 30 min, slides were incubated with anti-S100A8 (1/150) and anti-S100A9 (1/150); antibodies diluted in 1% BSA for 1 h at 37℃. After washing with PBS, slides were incubated with goat anti-mouse FITC (fluorescein isothicocyanate) 488 (ZSGB-BIO, ZF-0312) or rabbit anti-goat TRTIC (tetramethyl thodamine isothiocyanate) 555 (ZSGB-BIO, ZF-0317) conjugated secondary antibodies for 1 h at 37℃. The nuclei were stained using DAPI (4’, 6-diamidino-2-phenylindole). The targeted proteins were detected using confocal microscopy (ZEISS LSM700, Oberkochen, Germany) and a ZEISS LSM700 laser-scanning confocal microscope image system. Nonspecific IgG was used as a negative control.

**Tumorigenicity in nude mice**

Viable (5×10^6^) cells were mixed with HBSS, and six-week-old female nude mice were inoculated with them subcutaneously. BALB/C nude (nu/nu) mice were bought from the Peking University Laboratory Animal Center (Beijing, PR China). The tumors were washed with PBS and fixed with 4% buffered paraformaldehyde. All animal work was approved by the Ethics Committee of College of Life Science, Beijing Normal University (CLS-EAW-2014-006). All methods were carried out in accordance with relevant guidelines of that ethics committee. The animal experiment in this article did not use any anesthetic, and the method of sacrificed the animal was to break the neck.

**Tissue specimens**

Two hundred and fifty-seven SCC tissue specimens were obtained from seven cancer tissue arrays. One oral cavity SCC tissue array (OR601a), one skin cancer tissue array ( SK801b array), one cervical SCC tissue array (CR803), and four cancer tissue arrays ( No CC04-01-001, T271a，T022c, T124b) also including normal lung tissues, normal tongue tissues, normal esophageal tissues, normal cervical tissues and normal skin tissues were purchased from Xi’an Alenabio Company (Xian, China). All patients with cancer had received a pathological diagnosis and none had received prior therapy. All cancer tissues were obtained from surgically treated patients who gave their written informed consent to participate in this study and the Ethics Committee of the General Hospital of PLA Rocket Force (No. KY2015031) approved this protocol.

**ChIP**

Precleared chromatin of A431 was immunoprecipitated with a rabbit polyclonal antibody against YAP (Cell Signaling Technology, 4912), normal rabbit IgG (7074PZ, Cell Signaling Technology). Precipitated DNA was amplified with 2×EasyTaq SuperMix (TransGen Biotech). The primer for YAP binding site of the CYR61 promoter predicted by PROMO: 5- GGTA AGAT GCTT GTGG TTTG GC-3 (forward), and 5- CTTT TCCG TATG CGCT TTCG T-3(reverse). S100A8 promotor site primer: 5-AGGAAAGTGGGCAACAGAGC-3 (forward), and 5- TCTTGGAACTCACGGAGACCTA-3 (reverse). S100A9 promotor site primer: 5- CTCGGCTTTGGTAAGTGAGC-3 (forward), and 5- TTCTAGGAGGAGGATTTGGAAG-3 (reverse).

**Flow Cytometry**

The suspended and dense cultured cells were collected and washed twice with PBS buffer. Add 500μl binding buffer, 5μl AnnexinV-FITC and 5μl Propidium Iodide to each sample, and mix gently. Flow cytometry was used to determine the apoptosis proportion of the cells.

**Table S1. siRNA sequences**

| Genes name | siRNA sequences |
| --- | --- |
| YAP#A-sense | 5’GGUGAUACUAUCAACCAAATT3’ |
| YAP#A-antisense | 5’UUUGGUUGAUAGUAUCACCTT3’ |
| YAP#B-sense  YAP#B-antisense  LATS1#A-sense | 5’CUGCCACCAAGCUAGAUAATT3’  5’UUAUCUAGCUUGGUGGCAGTT3’  5’GAGCUGGAAAGGUUCUAAATT3’ |
| LATS1#A-antisense | 5’UUUAGAACCUUUCCAGCUCTT3’ |
| LATS1#B-sense | 5’GCAGCGUCUACAUCGUAAATT3’ |
| LATS1#B-antisense | 5’UUUACGAUGUAGACGCUGCTT3’ |
| MST1#A-sense | 5’GGACCUGCAUCAUGAACAATT3’ |
| MST1#A-antisense | 5’UUGUUCAUGAUGCAGGUCCTT3’ |
| MST1#B-sense | 5’GCUUCUCCUCCUGCCAUAUTT3’ |
| MST1#B-antisense | 5’AUAUGGCAGGAGGAGAAGCTT3’ |
| TEAD1#A-sense | 5’GCCACUGCCAUUCAUAACATT3’ |
| TEAD1#A-antisense | 5’UGUUAUGAAUGGCAGUGGCTT3’ |
| TEAD1#B-sense | 5’AUGGCCGAUUUGUAUACCGAATT3’ |
| TEAD1#B-antisense | 5’UUCGGUAUACAAAUCGGCCAUTT3’ |
| S100A8-sense | 5’CCAGGAGUUCCUCAUUCUGTT3’ |
| S100A8-antisense | 5’ CAGAAUGAGGAACUCCUGGTT3’ |
| S100A9-sense | 5’ GCAGCUGGAACGCAACAUATT3’ |
| S100A9-antisense | 5’ UAUGUUGCGUUCCAGCUGCTT3’ |

**Table S2. Primers used for qPCR**

| Genes name | Primers sequences |
| --- | --- |
| S100A8-sense | 5’GGGGAATTTCCATGCCGTCTA3’ |
| S100A8-antisense | 5’GGCTGCCACGCCCATCTTTA3’ |
| S100A9-sense  S100A9-antisense  CYR61-sense | 5’ATCAACACCTTCCACCAATACTCTG3’  5’AGGTTAGCCTCGCCATCAGC3’  5’GCTGCGAGGAGTGGGTCTGT3’ |
| CYR61-antisense | 5’GGGTTGTATAGGATGCGAGGCT3’ |
| CTGF-sense | 5’GCATCCGTACTCCCAAAATCTC3’ |
| CTGF-antisense | 5’CAGGGCACTTGAACTCCACC3’ |
| GAPDH-sense | 5’GAGTCAACGGATTTGGTCGT3’ |
| GAPDH-antisense | 5’GACAAGCTTCCCGTTCTCAG3’ |
| YAP-sense | 5’CCTCTATTTTGCTCTTCCTTGTCC3’ |
| YAP-antisense | 5’CCATCATCCAAACAGGCTCAC3’ |
| LATS1-sense | 5’CACCCTTCTTGGATACCACAGC3’ |
| LATS1-antisense | 5’CTGATTGACTCGTATGGAGGAACA3’ |
| MST1-sense | 5’TGCTTCTGACTCAATGCTTAGGG3’ |
| MST1-antisense | 5’TGGCTGCTCACGTTGTAGTGG3’ |
| TEAD1-sense | 5’TCGAGCAGCAGCGAGACCCAGACTC3’ |
| TEAD1-antisense | 5’TTACGAGGAAGAAGGCATTTTGAGG3’ |
| Keratin1-sense | 5’ TTATGGTCCTGTCTGCCCTCC 3’ |
| Keratin1-antisense | 5’ TGACTTGATTTGCTCCCTTTC 3’ |
| Keratin13-sense | 5’ TATGGAGGCGGCGTGAGC 3’ |
| Keratin13-antisense | 5’ CCAGGCGGTCGTTGAGGTT 3’ |
| TG1-sense | 5’ CCGTGGAGACCCAGTCAAT 3’ |
| TG1-antisense | 5’ CGGAATATCCCGTGCGTAG 3’ |
| Involucrin-sense | 5’ CAGCCAACTCCACTGCCTCCC 3’ |
| Involucrin-antisense | 5’ CTGCTCCTGTGGCTCCTTCTGC 3’ |

**Table S3.**  S100A8 and S100A9 expression in SCC tissues

| Groups | Type | No.  tested | S100A8 and S100A9  IHC staining score | | | | | No. Positive | | Percentage of positive  (No. positive/ No. tested) x100% | |
| --- | --- | --- | --- | --- | --- | --- | --- | --- | --- | --- | --- |
|  |  |  | 0 | 1 | 2 | 3 | 4 |  |  |  |  |
| Oral cavity | SCC | 51 | 2 | 4 | 24 | 12 | 9 | | 49 | | 96% |
| Skin | SCC | 42 | 2 | 7 | 11 | 15 | 7 | | 40 | | 95% |
| Esophageal | SCC | 4 | 0 | 1 | 3 | 0 | 0 | | 4 | | 100% |
| Lung | SCC | 90 | 11 | 31 | 29 | 9 | 10 | | 76 | | 84% |
| Cervical | SCC | 70 | 0 | 8 | 30 | 25 | 7 | | 70 | | 100% |

IHC staining score: 0, no positive cells; 1, <10% positive cells; 2, >10% and <50% positive cells; 3, >50% and <75% positive cells; 4, >75% positive cells.
