# Supplementary material for: The expression of S100A8/S100A9 is inducible and regulated by the Hippo/YAP pathway in squamous cell carcinomas
Source: BMC Cancer. 2019 Jun 17;19:597. doi: 10.1186/s12885-019-5784-0 (PMC6580480; doi:10.1186/s12885-019-5784-0)
Supplement: Supplementary file 2 — Figure S1. The Expression of S100A8 and S100A9 in HCC94 cells. Figure S2. Induction of S100A8 and S100A9 expression in A431 cells. Figure S3. Silencing effect of siRNAs in A431 cells. Figure S4. The expression of YAP and pYAP-S127 in xenografts. Figure S5. S100A8/A9 inhibit cell apoptosis induced by dense culture. Figure S6. YAP and p73 promote cell apoptosis induced by dense culture. Figure S7. Diagram to summarize S100A8 and S100A9 induction procedure. Figure S8. Diagram to summarize S100A8 and S100A9 induction procedure. (a) In normal adherent cultured cells, the Hippo pathway is in a closed state. YAP binding with TEAD in the nucleus, play a role in promoting cell proliferation and inhibiting of cell differentiation. YAP downstream protein ‘X’ binds to the promoter of S100A8 and S100A9, inhibiting S100A8 and S100A9 expression. (b) When SCC cells are detached or cultured in high density, the Hippo pathway are activated and nuclear YAP are decreased so that S100A8 and S100A9 lost the inhibitory effect on protein ‘X’, which leads to them induction. (DOCX 3844 kb) [file 12885_2019_5784_MOESM2_ESM.docx]

**
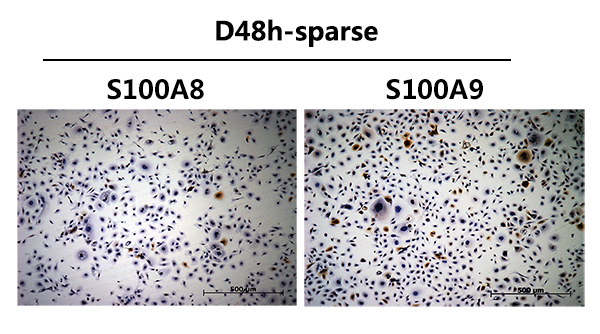
**

**Figure S1.** The Expression of S100A8 and S100A9 in HCC94 cells. Dense cells were reseeded at the pre-dense density, the expression of S100A8 and S100A9 were analyzed by immunohistochemical.

**
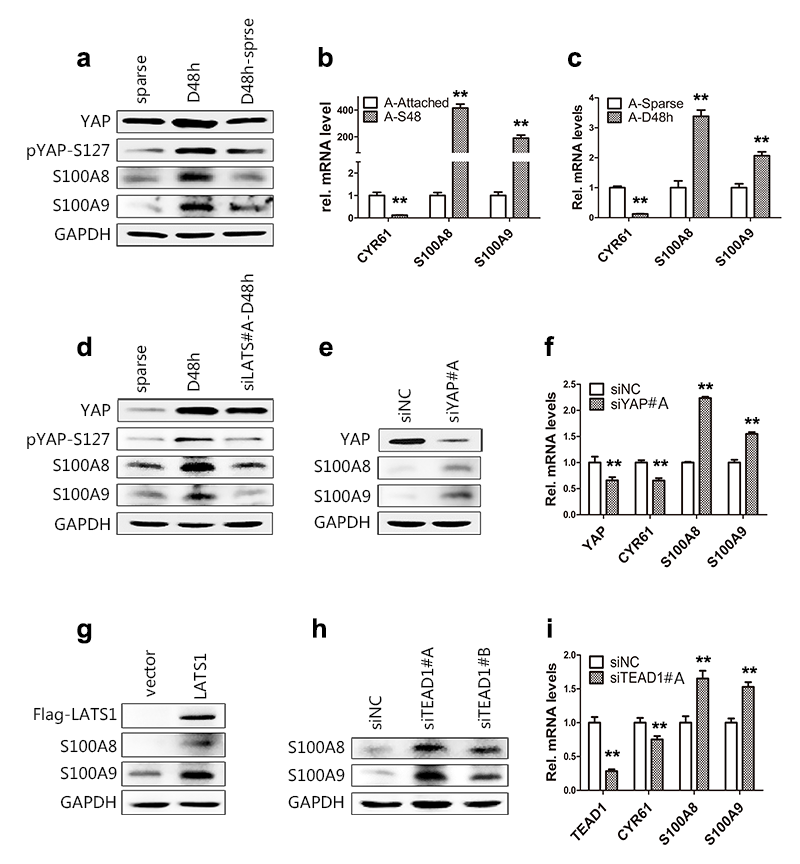
**

**Figure S2.** Induction of S100A8 and S100A9 expression in A431 cells. **(a)** Western blot analyses of S100A8, S100A9, YAP, pYAP-S127 in A431 cells. Cells were cultured densely for two days (D48h) and then relief from dense culture (D48h-sparse). GAPDH was used as a loading control. **(b, c)** The expression of S100A8, S100A9, CTGF and CYR61 were analyzed by qPCR in suspension or dense cultured A431 cells. Error bar, SD of three different experiments. **p<0.05, **p<0.01;* *t*-test. **(d)** LATS1 were knockdown by siRNAs in dense cultured A431 cells. Deletion of YAP in normal attached A431 cells, the expression of YAP, S100A8 and S100A9 were tested by western blot (**e**), and YAP, S100A8, S100A9 and CYR61 were detected by qPCR (**f**). Overexpression of LATS1 in normal attached A431 cells (**g**), anti-flag tag antibody was used to judge the transfection efficiency. TEAD1 was deleted by two specific siRNAs in A431 cells, the expression of S100A8 and S100A9 were detected by western blot (**h**), the expression of TEAD1, CYR61, S100A8 and S100A9 were detected by qPCR (**i**).

**
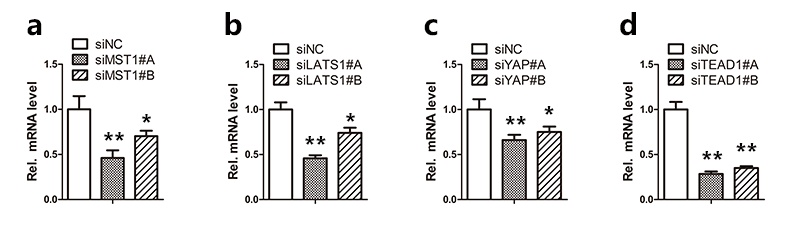
**

**Figure S3.** Silencing effect of siRNAs in A431 cells. Deletion of MST1 (**a**), LATS1 (**b**), YAP (**c**), TEAD1 (**d**) using siRNAs in normal attached A431 cells. The transfection efficiency of every two different specific siRNAs were detected by qPCR. Error bar, SD of three different experiments. **P < 0.05; *P < 0.01; t-*test*.*

**
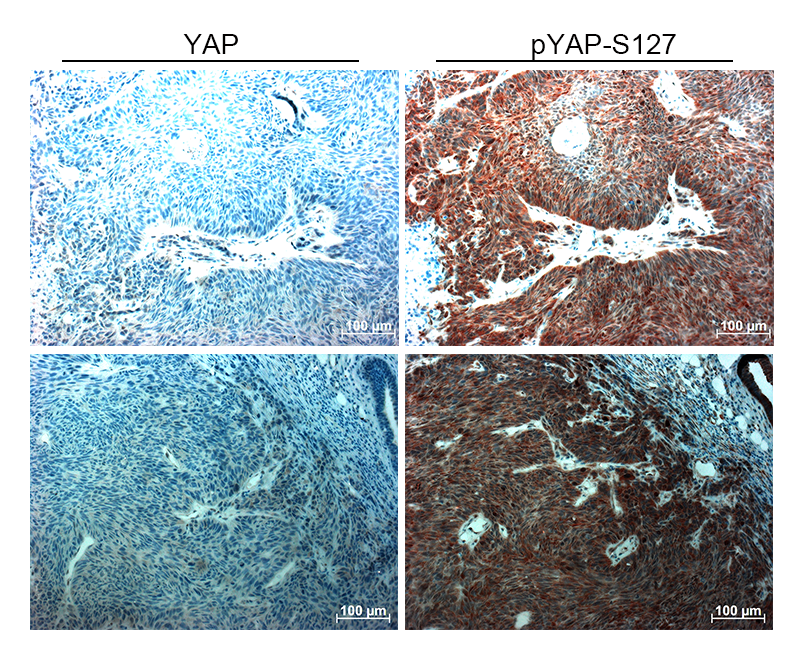
**

**Figure S4.** The expression of YAP, pYAP-S127 in xenografts. The staining of YAP, pYAP-S127 were examined in xenografts derived from A431 cells by immunohistochemistry in two consecutive sections.

**
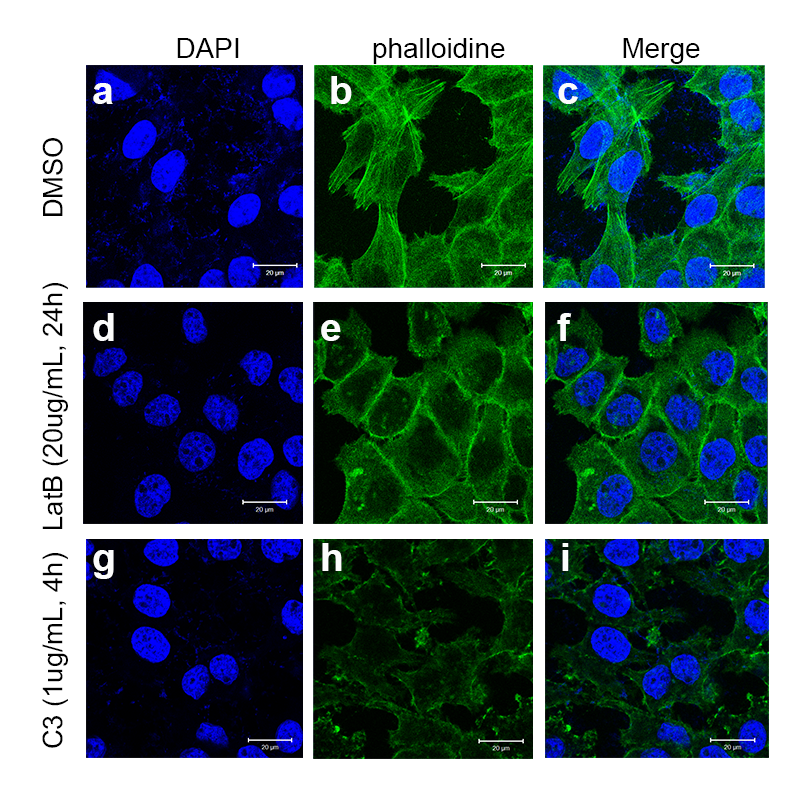
**

**Figure S5.** LatB and cytoD disrupt the F-actin cytoskeleton. Images showing the effect of actin inhibiting drugs LatB and C3 on the cytoskeleton in A431 cells.


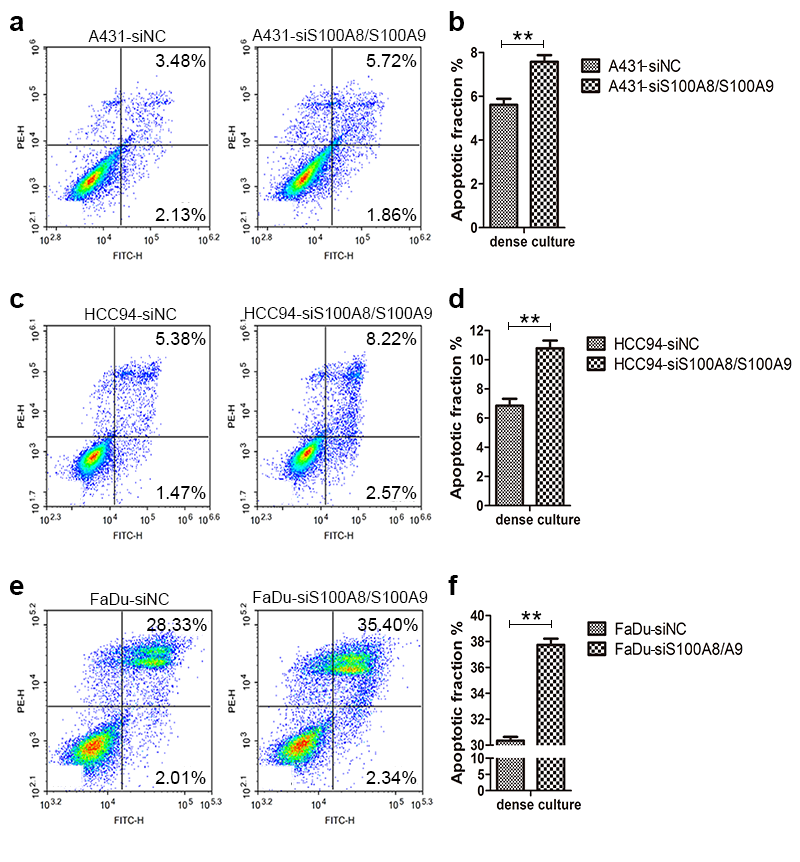


**Figure S6.** S100A8/A9 inhibit cell apoptosis induced by dense culture. A431 cells (**a, b**), HCC94 cells (**c, d**) and FaDu cells (**e, f**) were transfected with S100A8 and S100A9 specific siRNAs, 24 hours later cells were dense cultured 48 hours. The proportion of cell apoptosis was measured by Flow cytometry.


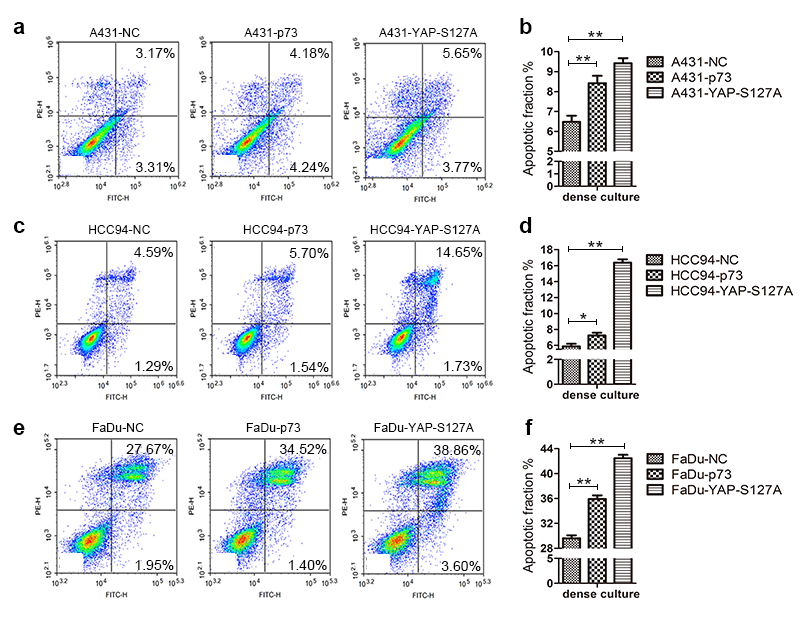


**Figure S7.** YAP and p73 promote cell apoptosis induced by dense culture. A431 cells (**a, b**), HCC94 cells (**c, d)** and FaDu cells (**e, f**) were transfected with p73 and YAP-S127A plasmids, 24 hours later cells were dense cultured 48 hours. The proportion of cell apoptosis was measured by Flow cytometry.


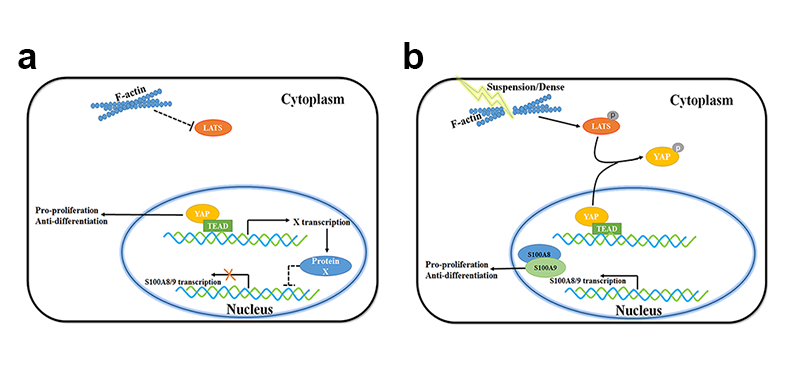


**Figure S8.** Diagram to summarize S100A8 and S100A9 induction procedure. **(a)** In normal adherent cultured cells, the Hippo pathway is in a closed state. YAP binding with TEAD in the nucleus, play a role in promoting cell proliferation and inhibiting of cell differentiation. YAP downstream protein ‘X’ binds to the promoter of S100A8 and S100A9, inhibiting S100A8 and S100A9 expression. **(b)** When SCC cells are detached or cultured in high density, the Hippo pathway are activated and nuclear YAP are decreased so that S100A8 and S100A9 lost the inhibitory effect on protein ‘X’, which leads to them induction.
